# Recent plastid replacement in *Karlodinium ballantinum* (Kareniaceae, Dinoflagellata) challenges the paradigms of endosymbiotic gene transfer

**DOI:** 10.1093/molbev/msag166

**Published:** 2026-07-07

**Authors:** Kacper Maciszewski, Kazuya Takahashi, Ryo Harada, Inga N Martinek, Takuro Nakayama, Mitsunori Iwataki, Yuji Inagaki, Elisabeth Hehenberger

**Affiliations:** Institute of Parasitology, Biology Centre, Czech Academy of Sciences, České Budějovice, Czechia; Institute of Parasitology, Biology Centre, Czech Academy of Sciences, České Budějovice, Czechia; Graduate School of Agricultural and Life Sciences, University of Tokyo, Tokyo, Japan; Institute for Comparative Genomics, Dalhousie University, Halifax, Nova Scotia, Canada; Institute of Parasitology, Biology Centre, Czech Academy of Sciences, České Budějovice, Czechia; Center for Computational Sciences, University of Tsukuba, Tsukuba, Japan; Graduate School of Agricultural and Life Sciences, University of Tokyo, Tokyo, Japan; Center for Computational Sciences, University of Tsukuba, Tsukuba, Japan; Institute of Parasitology, Biology Centre, Czech Academy of Sciences, České Budějovice, Czechia

**Keywords:** dinoflagellate, endosymbiotic gene transfer, horizontal gene transfer, phylogenetics, plastid endosymbiosis, plastid evolution

## Abstract

Plastids, the photosynthetic organelles of eukaryotes, arose via endosymbiosis of cyanobacteria by a eukaryotic host and were subsequently spread across eukaryotic diversity by additional endosymbioses. The process of plastid endosymbiosis is poorly understood, as most endosymbiotic events happened long ago. One group of microbial eukaryotes, the dinoflagellates, are characterized by their highly convoluted plastid evolution, particularly the family Kareniaceae, which have replaced their ancestral dinoflagellate plastid in most members with haptophyte plastids. To further explore the evolutionary history of kareniacean plastids, we obtained transcriptomic data from two representatives: *Gertia stigmatica* and *Karlodinium ballantinum*. We determined that *Gt. stigmatica* retained its ancestral plastid and that it is nested deep within the family Kareniaceae. Furthermore, the transcriptome shows no evidence of haptophyte plastid ancestry, indicating that a haptophyte plastid was likely never present. Conversely, *K. ballantinum* has abundant gene transfers originating from haptophytes, shared with other Kareniaceae. Surprisingly, *K. ballantinum*'s plastid genome is nearly identical to that of extant haptophyte *Gephyrocapsa huxleyi*, but we were unable to identify gene transfers from this current plastid across the transcriptome. We therefore conclude that (i) the phylogenomic position of *Gt. stigmatica* and its retention of the ancestral plastid support at least two independent plastid replacements in Kareniaceae, and (ii) *K. ballantinum* has replaced its plastid organelle twice, with the second replacement being as yet unaccompanied by endosymbiotic gene transfer. Phylogenomics of plastid genomes suggests that the unusually high plastid replacement rate in Kareniaceae might be caused by accelerated mutation of the plastid genome within the host.

Highlights

- *Gertia stigmatica* is the first known dinoflagellate in the family Kareniaceae without a haptophyte-derived plastid.
- *Karlodinium ballantinum* recently acquired its new plastid from a close relative of the haptophyte *Gephyrocapsa huxleyi*.
- Integration of a new plastid does not require large-scale endosymbiotic gene transfer.
- Plastids in Kareniaceae may require frequent replacement due to their accelerated evolution.

## Introduction

Endosymbiotic events, which led to the formation of mitochondria and plastids in eukaryotes, are among the evolutionary keystones that shaped the diversity of life on Earth (Keeling 2013; Archibald 2015). The establishment of a primary plastid derived from a cyanobacterial cell, which was engulfed by the ancestor of the Archaeplastida supergroup, was followed by multiple subsequent intereukaryotic endosymbioses ultimately leading to the spread of the photosynthetic organelles, termed complex plastids herein, across many lineages of eukaryotes (Keeling 2013; Burki 2017; Sibbald and Archibald 2020). Plastids of extant eukaryotes have been observed to perform a variety of metabolic functions besides photosynthesis, with their enzymatic machinery constituting locally encoded components, i.e. products of genes encoded in the plastid genome, as well as proteins imported from the host and encoded in its nucleus (Ponce-Toledo et al. 2019; Sibbald and Archibald 2020; Kolisko et al. 2024). Despite their location in the nuclear genome of the host, these genes are often of foreign origin, acquired either from the endosymbiont via endosymbiotic gene transfer (EGT), or even from other organisms via horizontal gene transfer (Novák Vanclová et al. 2020; Sibbald et al. 2021; Hehenberger and Burki 2024; Keeling 2024). In addition, for the organellar metabolism to be consistently maintained, the establishment of the novel organellar compartment requires the development of an efficient protein targeting and multi-membrane translocation mechanism (Keeling 2013). In complex plastids, proteins directed to the organelle are appended with bipartite N-terminal extensions, with a structure that includes a signal peptide (SP) and an organelle-specific transit peptide (TP), both often containing lineage-specific motifs (Waller et al. 2000; Patron et al. 2005; Patron and Waller 2007; Hirakawa et al. 2009; Keeling 2013).

Among plastid-bearing eukaryotes, dinoflagellates are known to have a particularly complex history of plastid evolution. Their ancestral red alga-derived complex plastids, the peridinin plastids (named after the specific pigment), have undergone frequent reduction to non-photosynthetic forms (and in rare cases have even been lost completely) or have been replaced several times by plastids of different algal origins (Gornik et al. 2015; Waller and Kořený 2017; Cooney et al. 2024). A showcase for convoluted plastid evolution can be found in the dinoflagellate family Kareniaceae. Early studies of this family (genera *Karenia*, *Karlodinium,* and *Takayama*) revealed that the peridinin plastid has been replaced by a haptophyte-derived plastid (Tengs et al. 2000; Gabrielsen et al. 2011). In a more recent study, however, it has been demonstrated that the kareniacean Ross Sea Dinoflagellate (RSD) potentially houses two distinct types of plastids—permanent, albeit reduced, non-photosynthetic peridinin plastids, and transient photosynthetic kleptoplasts of haptophyte origin (Hehenberger et al. 2019). This study also suggested the presence of remnant peridinin plastids in the other members of the Kareniaceae. Interestingly, recent discoveries have shown that there is a kareniacean lineage that still carries a photosynthetic peridinin plastid (genus *Gertia*, represented by two described species: *Gertia stigmatica* [Takahashi et al. 2019] and *Gertia obesa* [Takahashi et al. 2025]). Moreover, the phylogenetic origins of several plastid-encoded genes imply that two lineages, *Karlodinium armiger* and *Takayama helix*, have replaced the haptophyte plastid with another plastid of a different haptophyte origin (Novák Vanclová et al. 2024). Finally, the recently described kareniacean genus *Shimiella* also utilizes kleptoplasts; however, in contrast to its close relative RSD, its transient plastid donors are cryptophytes, instead of haptophytes (Ok et al. 2021). These observations suggest that the evolutionary history of plastids in Kareniaceae is likely even more complex than anticipated in previous studies.

Thus far, several models explaining the distribution of plastids of diverse origins in Kareniaceae have been proposed (Takahashi et al. 2019; Novák Vanclová et al. 2024); however, cultures and data for a large part of the kareniacean diversity are lacking, which prevents a better understanding of the mechanisms underlying the observed plastid distribution. To shed new light on the evolution of plastids of kareniacean dinoflagellates, we present an in-depth analysis of the identity and evolutionary origins of the plastid organelles and metabolic components in two representatives of Kareniaceae: *Gt. stigmatica* and the newly cultivated *K. ballantinum*, whose genomic and/or transcriptomic data have not been previously available. Furthermore, to deepen our understanding of the characteristics of replaced plastids and to remediate the scarcity of genomic data from kareniacean plastids, we obtained a near-complete plastid genome sequence of *T. helix*—the first genome from a haptophyte-derived plastid outside of the genus *Karlodinium*. This enabled us to perform a plastid-based phylogenomic analysis with broader taxon sampling and to estimate the rate of evolution of plastid genomes in different branches of Kareniaceae.

## Results and discussion

### Plastid genome of *K. ballantinum* points toward a very recent replacement

**Figure 1:**
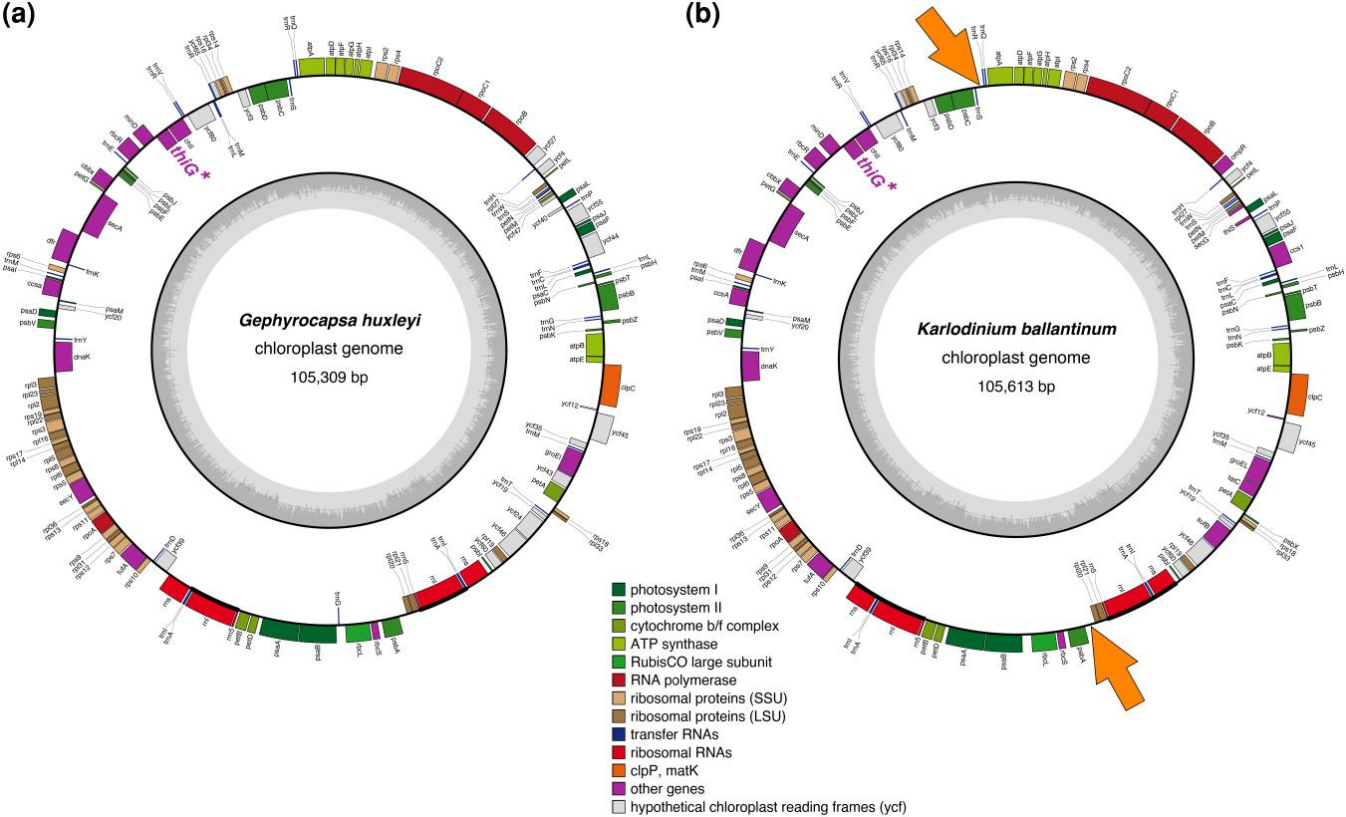
Plastid genome maps of a) *Gc. huxleyi* (NCBI accession no. AY741371) and b) *K. ballantinum* (this study; accession no. PZ117046). Arrows indicate the intergenic regions expanded in the *K. ballantinum* genome compared to the *Gc. huxleyi* genome and asterisks indicate the *thiG* gene present in both plastid genomes.

Initial automatic annotation revealed that the gene complement and order in the plastid genome (ptDNA) of *K. ballantinum* are identical to that of the haptophyte *Gephyrocapsa huxleyi* (GenBank accession no. AY741371), while being substantially different from the only available kareniacean ptDNA of *Karlodinium veneficum* (GenBank accession no. JN039300). Following manually curated annotation, we observed that the sequence identity between the plastid genomes of *K. ballantinum* and *Gc. huxleyi* is extremely high, with 98.9% overall identity on the nucleotide level, ranging from 98.34% to 100% in protein-coding genes. For comparison, the plastid genome of *K. ballantinum* has 97.2% identity on the nucleotide level with the plastid genome of *Gephyrocapsa oceanica*, the only other species of this genus for which a nuclear transcriptome is available (necessary for subsequent analyses). The plastid genome map comparison is shown in Fig. 1. The only noticeable structural difference between the two plastid genomes is the expansion and divergence of two short intergenic regions (*psbA–rpl20* and *atpA–psbC*) in *K. ballantinum*, which accounts for the 304 bp difference in size between the two genomes.

Thus, with very few signs of genetic divergence from its haptophyte donor, it is most likely that the plastid replacement in *K. ballantinum* is a very recent event. This high similarity might also indicate that the plastid found in *K. ballantinum* is a *Gc. huxleyi*-derived kleptoplast. However, the *K. ballantinum* strain from which the data were obtained has been maintained for over 5 years as a clonal monoeukaryotic culture, derived from hand-picked single cells and without observed changes in plastid number and morphology, suggesting a stably integrated plastid.

### Transcriptome of *K. ballantinum* bears little trace of *Gephyrocapsa-*derived genes outside of the plastid genome

**Figure 2:**
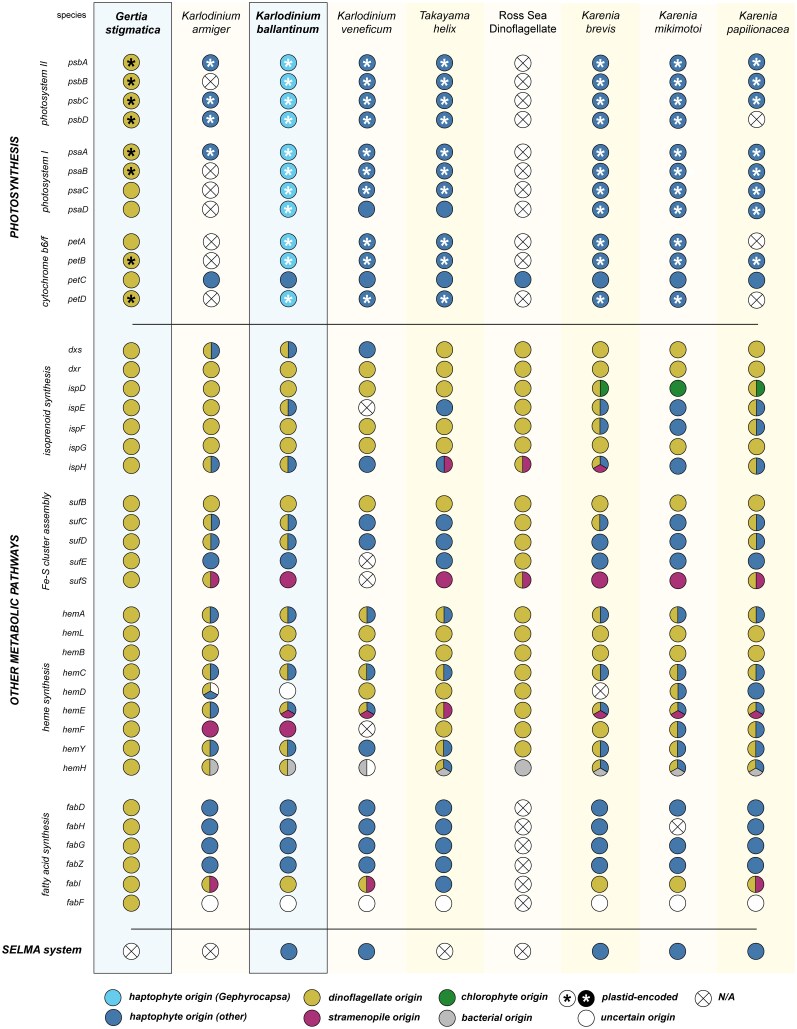
Schematic depiction of evolutionary origins of plastid metabolic and plastid import (SELMA) enzymes in Kareniaceae. Each circle represents an enzyme, colored by its evolutionary origin as determined by phylogenetic reconstructions. Sectioned circles indicate more than one evolutionary origin for a given enzyme.

Although the generally accepted paradigm states that the acquisition of a plastid organelle is associated with EGT, the available data on the extent of EGT in lineages with complex plastids show tremendous diversity, with estimated numbers of transferred genes ranging from zero to several hundred (Hehenberger and Burki 2024). To search *K. ballantinum* for EGTs from the donor of its current plastid (likely a close relative of *Gc. huxleyi*), we investigated the evolutionary origins of a set of plastid-directed proteins (termed “plastid-targeted protein set” here) known to contain genes derived from EGT in Kareniaceae. Unexpectedly, we identified all haptophyte gene transfers in *K. ballantinum* to be shared with other Kareniaceae and not related to *Gc. huxleyi* (Fig. 2 and “Plastid Metabolic Pathways and Plastid Import Phylogenies” in the FigShare repository at https://doi.org/10.6084/m9.figshare.31541731). In addition, gene transfers in *K. ballantinum* from other sources (e.g. stramenopiles) have a shared origin with several kareniacean representatives. Finally, for some gene products, the ancestral dinoflagellate version is targeted to the plastid in all Kareniaceae, including *K. ballantinum*. Together, these shared evolutionary origins not only confirm the presence of a prior haptophyte plastid in *K. ballantinum* but also indicate that the plastid-targeted proteins still present from the former plastid are being recycled for usage in its new haptophyte plastid.

To investigate *K. ballantinum* for potential EGTs outside of this set of known plastid-targeted proteins, we performed a transcriptome-wide screening by reconstructing the ortholog groups for every expressed gene in *K. ballantinum*, using a curated database of 72 dinoflagellate and 31 haptophyte transcriptomic datasets. Since only two eukaryotic groups were included in this initial analysis, the obtained phylogenetic trees will hereby be referred to as local trees.

Among 254,267 local ortholog trees, 8,774 contained both (i) at least one *K. ballantinum* sequence and (ii) at least five haptophyte sequences—a restriction we set to identify gene transfers from haptophytes with confidence. These trees were subject to screening, both manually and using a tree-sorting script, to identify monophyletic clades of *K. ballantinum* and members of the haptophyte family Noelaerhabdaceae, to which *Gc. huxleyi* belongs. The two screening methods yielded fully congruent results, enabling successful identification of 64 trees containing such clades, which were selected for further analysis. Twenty-seven of these trees contained sequences from *K. ballantinum* and *Gc. huxleyi* in a sister relationship. All 27 of these putative *Gc. huxleyi*-derived transcripts in *K. ballantinum* were further identified as products of genes encoded in the plastid genome, including, among others, photosystem (PS) I and II components, ATP synthase components, plastid ribosomal proteins, and the large RuBisCO subunit (see, e.g., proteins indicated in light blue in Fig. 2).

In the remaining 37 phylogenies, a *K. ballantinum* sequence was placed as sister to a clade containing only sequences from Noelaerhabdaceae (represented in our database by *Gc. oceanica,* two strains of *Gc. huxleyi,* and the strain Isochrysidales sp. CCMP1244). For this group, the 37 putative transcripts of potential noelaerhabdacean origin in *K. ballantinum* were extracted and used as queries in a BLASTP search against our full eukaryotic database to reconstruct global ortholog trees. Five trees could not be reconstructed due to the lack of identifiable BLAST homologs to the query sequence, even at an *e-*value cutoff of 0.001—in these cases, it is likely that the trees were reconstructed based on orthogroups consisting of erroneously assigned, non-homologous sequences. Among the 32 global trees that we were able to reconstruct, 26 resolved with the *K. ballantinum* sequence branching either inside dinoflagellates or with non-noelaerhabdacean haptophytes, contradicting a transfer from the current plastid. On the remaining eight global trees, a topology with *K. ballantinum*, *Gc. huxleyi,* and other Noelaerhabdaceae forming a clade was observed; however, *K. ballantinum* branched either with low support as sister to *Gc. oceanica* (one candidate) or as sister to the entire Noelaerhabdaceae family (seven candidates) (see “*K. ballantinum* Gene Transfer Phylogenies” in the FigShare repository at https://doi.org/10.6084/m9.figshare.31421609 and Table S1). This indicates that these latter seven genes were likely not transferred from the current *Gc. huxleyi*-like plastid but may have been obtained from former associations with noelaerhabdacean-related haptophytes. That left one potential candidate as a transfer from the current plastid; however, this candidate is unlikely to be an EGT because of its relatively long branch length and the lack of evidence for plastid function. The absence of clear candidates for EGT from *Gc. huxleyi* in a transcriptome-wide search further supports the idea that the current plastid in *K. ballantinum* is maintained in large part by the machinery already present before its acquisition.

We cannot exclude the possibility that the transfer of genes from *Gc. huxleyi* to *K. ballantinum* is still ongoing. Although the data for *K. ballantinum* were obtained from a long-term culture without any prey, it may still feed in its natural environment, which would allow gene transfer not only from the current plastid but also from free-living *Gephyrocapsa* nuclear genomes. Conversely, the lack of nucleus-to-nucleus gene transfer in *K. ballantinum* may have a different mechanistic explanation related to its feeding behavior. Dinoflagellates, including Kareniaceae, have been shown to feed via myzocytosis, among several other mechanisms (Waller and Carruthers 2024; Ok and Jeong 2025). This mode of feeding, in which the predatory cell sucks out the prey cell content via a specialized feeding structure, would allow the selective acquisition of plastids, inherently preventing gene transfer from the prey nucleus. Nonetheless, our observations indicate that at least in this case of a repeated replacement of likely similar plastids, massive EGT is not necessary for initial establishment of the plastid.

### Transcriptomic data reveal no trace of the haptophyte plastid in *Gt. stigmatica*

**Figure 3:**
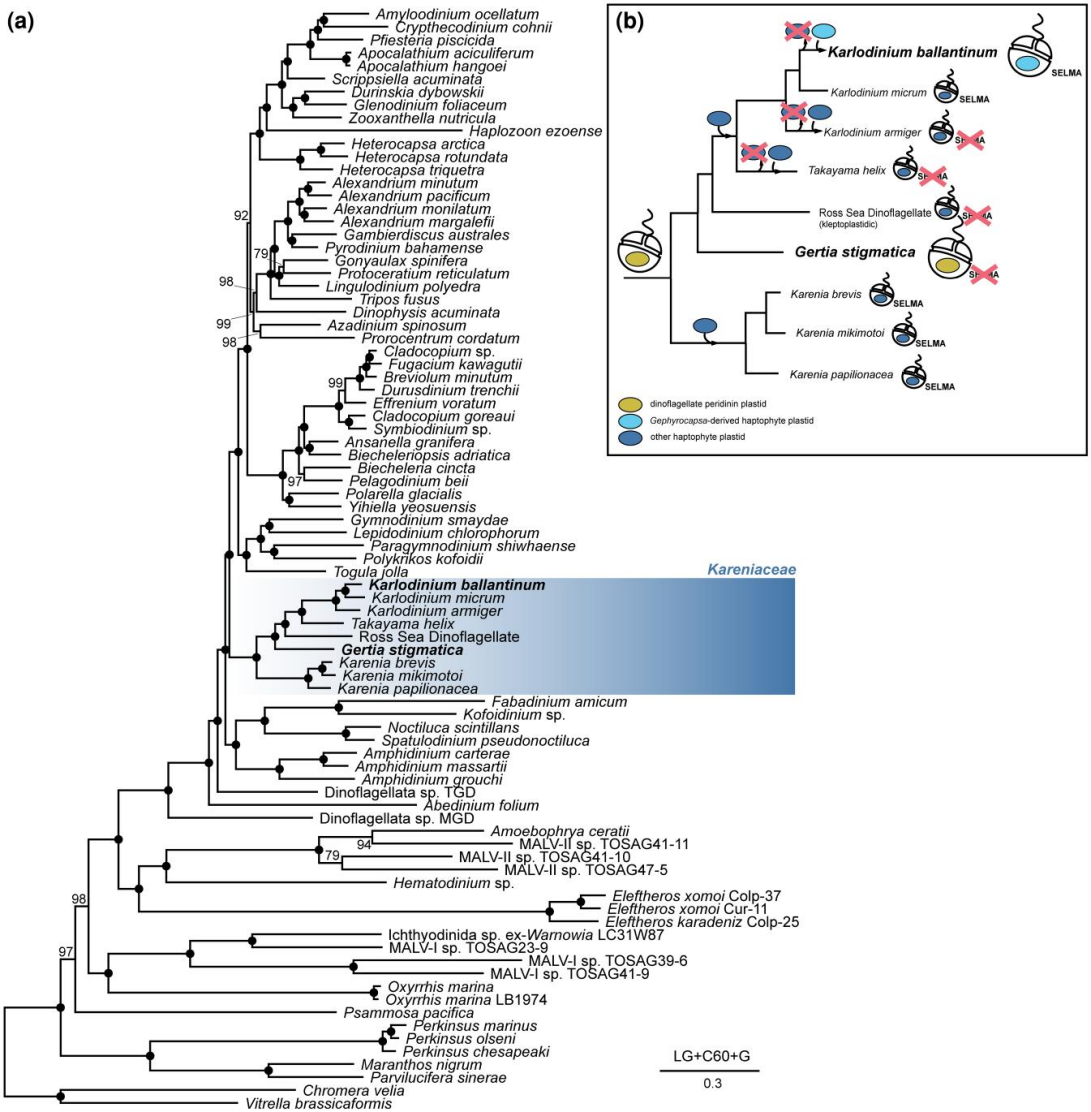
Phylogenomic reconstruction of dinoflagellates and schematic phylogeny of Kareniaceae. a) ML analysis of a 228 multi-protein alignment (LG + C60 + G plus -mwopt) of dinoflagellates plus perkinsids + chromerids as outgroup. Kareniaceae are indicated by a blue box, and taxa shown in bold indicate original data obtained in this study. Non-parametric bootstrapping was performed in 500 replicates, and dots at the nodes indicate full statistical support. b) Plastid evolution and absence/presence of the SELMA system in Kareniaceae. Yellow plastids represent ancestral dinoflagellate peridinin plastids, dark blue plastids represent haptophyte-derived plastids, and the light blue plastid represents the relatively recently replaced plastid of *K. ballantinum*.

To achieve a deeper understanding of the evolution of kareniacean plastids, we additionally studied the only kareniacean lineage that was shown to retain a photosynthetic peridinin plastid, *Gt. stigmatica* (Takahashi et al. 2019). To unambiguously place *Gt. stigmatica* within the tree of dinoflagellates, we added 81 dinoflagellate taxa to the 8 dinoflagellates (including *Perkinsus marinus*) present within the original PhyloFisher (Tice et al. 2021) data matrix and reconstructed a phylogenomic tree. As shown in Fig. 3a, *Gt. stigmatica* branches with absolute support within Kareniaceae, at the base of the *Karlodinium–Takayama–*RSD clade. This topology is congruent with the phylogeny of the large ribosomal RNA subunit reconstructed in the original description of the genus *Gertia* (Takahashi et al. 2019). As discussed by Takahashi et al. (2019), although *Gertia* has been shown to carry the ancestral peridinin plastid, its deeply nested position within the Kareniaceae suggests that, like in its kareniacean relatives, an association with a haptophyte plastid might have taken place in its past. Such an association potentially left behind traces in the form of a haptophyte-derived cryptic plastid and/or retargeting of haptophyte-derived proteins to the peridinin plastid. To verify this hypothesis, we first investigated the evolutionary origins of the “plastid-targeted protein set” in *Gt. stigmatica*, along with their N-terminal plastid-targeting peptides. We did not detect any haptophyte plastid footprint in *Gt. stigmatica* using this approach (Fig. 2), as none of the components of the investigated plastid metabolic pathways have a haptophyte phylogenetic signal or an N-terminal targeting sequence that is typically found in dinoflagellates with haptophyte plastids, i.e. a hydrophobic SP, followed by an RR motif, marking the beginning of the TP, which is enriched in serine, threonine, and basic amino acids (Patron et al. 2006; Lewis et al. 2024). Likewise, despite being common in the investigated pathways among all other Kareniaceae, gene transfers from non-haptophyte sources, such as stramenopiles, were not detected in *Gt. stigmatica* (Fig. 2). It is unlikely that these observations reflect missing data, as we successfully identified all or nearly all components of several plastid metabolic pathways in each of the analyzed datasets, with their completeness estimated between 73.3% (*K. veneficum*) and 86.3% (RSD) (Table S2; *Gt. stigmatica* = 75.2%).

As was done for *K. ballantinum*, we broadened our search for a haptophyte footprint in *Gt. stigmatica* to the whole transcriptome to identify potential plastid-associated gene transfers that function in pathways not covered by our specific search. Among all reconstructed orthogroup trees (*n* = 254,267 reconstructed global trees, as outlined above), over 1,200 *Gt. stigmatica* sequences were recovered as forming a monophyletic clade with haptophytes. Automated sorting and manual inspection revealed only 36 global trees that support potential transfers, with the *Gt. stigmatica* sequence branching within haptophyte sequences (see “*Gt. stigmatica* Gene Transfer Phylogenies” in the FigShare repository at https://doi.org/10.6084/m9.figshare.31421609). This number stands in stark contrast to the significantly higher estimates for haptophyte gene transfers in Kareniaceae with permanent haptophyte plastids, which range from 345 to 1,089 (Novák Vanclová et al. 2024). Out of these 36 trees, 25 have a topology that suggests a gene transfer from a haptophyte donor to *Gt. stigmatica* specifically but not to any other kareniacean. In the remaining 11 trees, the *Gt. stigmatica* + haptophyte clade contained at least one sequence of a haptophyte plastid-bearing kareniacean. We were unable to computationally determine the subcellular localization for any of these 36 gene transfer candidates in *Gt. stigmatica,* as we could not identify a known plastid-targeting N-terminal extension for the 22 homologs with complete coding sequences, and the remaining 14 were missing the N-terminus.

Most of the 36 transfer candidates in *Gt. stigmatica* contain known protein domains (5 of the 36 yielded no hits to any domains): 26 sequences include functional enzymatic domains, while a further 5 had only identifiable transmembrane domains. Transcript no. 115625 carries several putative functional domains, including a glyceraldehyde 3-phosphate dehydrogenase domain, which can also function in plastids (Muñoz-Bertomeu et al. 2009). Similarly, transcript no. 78234 was identified as a putative glutathione S-transferase—a protein with numerous identified homologs in the model plant *Arabidopsis thaliana*, some of which are plastid-targeted (Dixon and Edwards 2010). However, evidence supporting the plastid localization of these two proteins in *Gt. stigmatica* could not be found in their sequences as outlined above. Overall, none of the identified transfer candidates in *Gt. stigmatica* could be associated with a function specific to plastid metabolism (Table S1).

The collective results from our investigation of the *Gt. stigmatica* transcriptome do not provide any strong evidence of plastid-associated gene transfer from a haptophyte (or any other algae), and consequently, no indication for a plastid other than the peridinin plastid being present in this organism in the past. Nevertheless, an ecological association with a haptophyte may have taken place at some point in its evolutionary history, which has left small traces in the transcriptome as persisting horizontally transferred genes probably unrelated to a plastid endosymbiosis. According to published studies (reviewed in Ok and Jeong 2025), *Gt. stigmatica* is one of only four described non-mixotrophic kareniacean species; however, that does not exclude the possibility that *Gt. stigmatica* or its ancestors used to be mixotrophic predators, or that we have yet to discover the circumstances under which this lineage feeds (Jeong et al. 2010). In addition, as described previously, kareniaceans with permanent haptophyte plastids still retain many plastid-targeted proteins of dinoflagellate origin, in several cases even though haptophyte-derived homologs are also present, leading to the coexistence of two (or more) redundant copies (Fig. 2) (Hehenberger et al. 2019). Such redundancy is absent in *Gt. stigmatica*, suggesting that either haptophyte genes were never present in this organism or that they were acquired and subsequently lost from its genome. We consider the latter scenario rather unlikely, as the selective force leading to the elimination of redundant homologs would have to act exclusively on the haptophyte genes in *Gt. stigmatica*, but not on the peridinin plastid-associated genes. No such specific elimination of redundancy (e.g., of the peridinin plastid genes) can be observed in any of the other Kareniaceae, with every lineage showing some degree of redundancy in the investigated pathways and without any discernable pattern. Therefore, we maintain that the most likely scenario is that the lineage leading to *Gt. stigmatica* was not subject to extensive EGT from haptophytes and likely did not support a permanent haptophyte-derived plastid in its evolutionary history.

In conjunction with the nested position of *Gt. stigmatica* within Kareniaceae, this strongly indicates independent plastid establishment in the *Karenia* and *Karlodinium*/*Takayama*/RSD clades or, alternatively, its single establishment in one of the lineages mentioned above and subsequent acquisition by the other via serial endosymbiosis, as proposed by Novák Vanclová et al. (2024). Taken together with the evidence of repeated plastid replacement in *K. ballantinum*, *K. armiger,* and *T. helix*, our observations support a model where the ancestor of the Kareniaceae possessed a peridinin plastid that has since been replaced at least two times with a haptophyte plastid, which itself has been supplanted in at least three lineages (Fig. 3b).

### Insights from *K. ballantinum* and *T. helix* show divergent tendencies in twice-replaced plastids

Haptophyte plastids require a different membrane trafficking machinery than peridinin plastids due to the presence of an additional surrounding membrane: peridinin plastids are surrounded by three membranes, while most other complex plastids, including the plastids of haptophytes, possess four membranes (Burki 2017). Trafficking across three membranes in peridinin plastids uses the TIC/TOC protein complexes for translocation across the first (innermost) and second membrane (Lewis et al. 2024), while translocation across the outermost membrane utilizes the host's Golgi-mediated vesicle system (Nassoury et al. 2003). In addition to TIC/TOC, haptophyte plastids possess the SELMA translocon—a divergent duplicate of the endoplasmic reticulum-associated degradation machinery—which facilitates protein translocation across the third plastid membrane (Stork et al. 2013). While not found in peridinin plastid-bearing dinoflagellates, the SELMA system has been identified in haptophyte plastid-bearing kareniaceans, as it was likely necessary for the establishment of a haptophyte plastid (Lewis et al. 2024). A recent study, though, has shown that the SELMA machinery is absent in two kareniacean lineages that probably independently replaced their haptophyte plastid for a second time, *K. armiger* and *T. helix*, suggesting their plastids are only surrounded by three membranes, as in peridinin plastids (Lewis et al. 2024; Novák Vanclová et al. 2024). However, we identified all of the haptophyte-derived SELMA components in the transcriptome of *K. ballantinum* (Fig. 2 and Fig. S1). This suggests that the twice-replaced *K. ballantinum* plastid is likely surrounded by four membranes, similar to the once-replaced plastids of *Karenia* spp. and *K. veneficum*, as well as the original plastids of haptophytes, and not three membranes, like the twice-replaced plastids of their close relatives *K. armiger* and *T. helix* (as indicated by the presence/absence of the SELMA system in Fig. 3b) (Lewis et al. 2024; Novák Vanclová et al. 2024). It therefore seems that the number of membranes surrounding a plastid does not correspond to the recency of a given endosymbiotic relationship, rather it is likely determined during the process of plastid integration. This process can evidently lead to radically different outcomes in terms of organellar architecture, even in recently diverged hosts that have taken up closely related symbionts.

Peridinin plastid–bearing dinoflagellates perform RNA editing of mitochondrial and plastid gene transcripts (Lin 2024). The editing of plastid genome transcripts has also been observed in the green algal plastid–bearing dinoflagellate *Lepidodinium* spp. and the haptophyte plastid-bearing kareniaceans *Karenia mikimotoi* and *K. veneficum* (Dorrell and Howe 2012; Jackson et al. 2013; Matsuo et al. 2022; Lin 2024). To identify whether this mechanism also operates in kareniaceans with twice-replaced plastids, we compared the sequences derived from transcriptomic datasets and plastid genome assemblies of *K. ballantinum* and *T. helix*. Of note, the *T. helix* transcriptome stems from a publicly available dataset and is based on a strain obtained from culture collection (Novák Vanclová et al. 2024), while the plastid genome was generated from an environmental strain of *T. helix*, which had been established (and has since perished) as part of this investigation. We therefore expect a certain extent of divergence between these two datasets, independent of the presence of RNA editing. Our nearly complete assembly of the plastid genome of *T. helix* (Fig. S2) enabled the recovery of 79 full protein-coding gene sequences, out of which 8 ptDNA-specific genes (i.e. only those functionally related to photosynthesis and without known eukaryotic homologs acting in subcellular locations other than the plastid) were selected for comparative analysis of nucleotide sequences with their transcriptome-derived counterparts. We observed the genome-derived and transcriptome-derived sequences to be tremendously divergent, with pairwise identity ranging between 63.3% and 78.0% (Table S3). In comparison, transcript editing has been found to account for only up to 6.7% divergence between the gene and transcript sequences in *K. mikimotoi* (Dorrell and Howe 2012). As mentioned above, some of this divergence might reflect differences between the two *T. helix* strains used for data generation. We therefore performed mapping of the *T. helix* transcriptomic reads to the *T. helix* plastid genome to test whether reads derived from DNA putatively contaminating the transcriptomic data (commonly observed in RNA sequencing data) map to the plastid genome assembly. Indeed, we found reads mapping (with a coverage from 10 to 68,000 reads) to the eight ptDNA-specific genes analyzed above, with pairwise identities ranging from 94.0% to 97.2% (Table S3). Since this observation is based on putative contamination and we cannot exclude the possibility that the observed differences are caused by other factors, we are unable to conclusively resolve whether *T. helix* performs RNA editing. In addition, we analyzed another transcript-modification process typically observed in dinoflagellate plastids, the addition of 3'-poly(U) tails (Dorrell and Howe 2015); however, none of the 42 complete transcripts of plastid-encoded genes (out of 53 identified in the transcriptomic data) were found to carry 3'-poly(U) tails. Therefore, whether *T. helix* performs RNA editing or other transcript modifications remains an open question that will have to be specifically targeted by future studies.

When we performed this comparative analysis on eight genes and transcripts from *K. ballantinum* (for which all datasets were obtained from the same isolate), we found that all analyzed sequence pairs were 100% identical (Table S3), indicating that the transcripts of genes encoded by this recently acquired plastid likely do not undergo any RNA editing. This suggests that, unlike the number of plastid membranes, RNA editing may correlate with plastid age and may therefore not begin happening in a new plastid organelle in the initial stages of its establishment.

### Plastid replacement may be adaptive, but a rescue mechanism hypothesis seems more universal

The recent plastid replacements in *K. ballantinum*, *K. armiger*, and *T. helix*—lineages that already replaced their ancestral dinoflagellate plastid once—raise the question whether such frequent replacements of this organelle provide any advantage to the host. In the case of *K. ballantinum*, without meaningful EGT, the only source of evolutionary novelty for the host came with the *Gc. huxleyi*-like plastid genome. When comparing this genome with the available genomic and transcriptomic data of other kareniacean lineages, we found only one gene, *thiG*, in the *Gc. huxleyi*-like plastid genome (Fig. 1) that is absent from both plastid genomes and nuclear transcriptomes of all other Kareniaceae. *thiG* encodes thiazole synthase, which enables the synthesis of thiamine (vitamin B1) from 1-deoxy-D-xylulose 5-phosphate (DXP) (Park et al. 2003; Hazra et al. 2009), an intermediate compound in the plastidial isoprenoid biosynthesis pathway (Lange et al. 2000). Thiamine is a cofactor of enzymes involved in central carbon metabolism and is indispensable for nearly all cellular life (Tang et al. 2010; Paerl et al. 2018); however, it is synthesized de novo from DXP predominantly by bacteria and only some red plastid-bearing eukaryotes (e.g., most red algae, as well as some ochrophytes and haptophytes), while plants and green algae utilize a different biosynthetic pathway (Raschke et al. 2007; Guan et al. 2014). Other eukaryotes (e.g., opisthokonts and most cryptophytes, but also most dinoflagellates) are auxotrophically dependent on its uptake from external sources (Tang et al. 2010). Consequently, thiamine has been described as a growth-limiting factor for a variety of organisms in oceanic ecosystems (Fridolfsson et al. 2020), including Kareniaceae, as demonstrated by the in vivo experiments on their growth requirements (Tang et al. 2010).

The presence of *thiG* (and most likely the capacity for thiamine synthesis) is patchily distributed among haptophytes, as it is present in the sequenced plastid genomes of *Prymnesium, Chrysochromulina, Isochrysis, Tisochrysis,* and *Gephyrocapsa* strains, but not *Pavlova, Diacronema, Pavlomulina,* or *Phaeocystis* strains (Table S4). Moreover, the lack of *thiG* in available kareniacean transcriptomes and the plastid genome of *K. veneficum* suggests that the donor of the original kareniacean haptophyte plastids probably did not carry this gene either. Therefore, the gain of plastid-encoded thiazole synthase may provide *K. ballantinum* with an evolutionary innovation that makes replacement of the whole plastid organelle advantageous. This explanation might also apply to certain other Kareniaceae that replaced their haptophyte-derived plastids with alternative haptophyte-derived plastids. As *thiG* is absent from all three sequenced plastid genomes of *Phaeocystis* spp. (Smith et al. 2014; Song et al. 2021), its absence in the *Phaeocystis-*derived plastid genome of *T. helix* is not surprising. However, plastid genomes of *Prymnesium parvum* strains and the transcriptome of *Prymnesium polylepis* all contain *thiG* homologs, which may have been acquired by *K. armiger* along with its current plastid; however, we could not identify this gene in the published transcriptome of *K. armiger,* and thus far, no genomic data from this dinoflagellate is available (Jian et al. 2024; Novák Vanclová et al. 2024).

**Figure 4:**
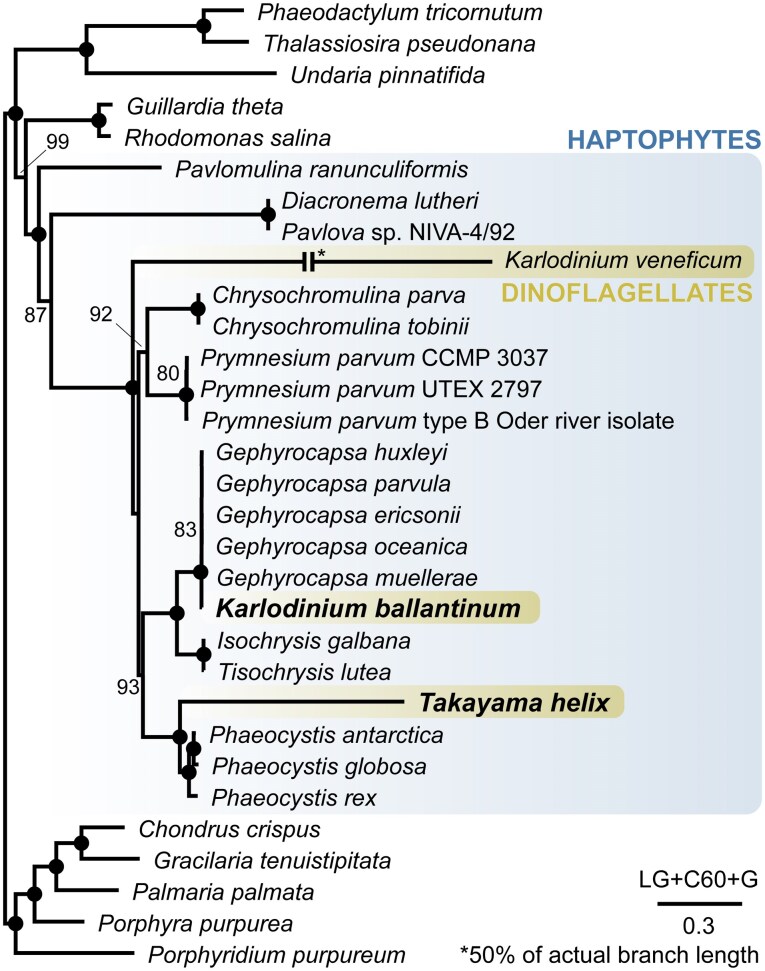
Plastid phylogenomic tree of red algae and red algal plastid-bearing lineages. ML analysis of a 96 multi-protein alignment (LG + C60 + G plus -mwopt) generated from proteins predicted from plastid-encoded genes. Haptophytes are indicated by a blue box, and haptophyte plastid-bearing dinoflagellates are indicated with yellow boxes. Non-parametric bootstrapping was performed in 500 replicates, and dots at the nodes indicate full statistical support, while support values below 70 are not shown.

An alternative possibility is that repeated plastid replacement is not entirely advantageous per se, but that it rather functions as a rescue mechanism. In single-protein phylogenies of plastid-encoded genes (see e.g. PS I and II phylogenies in “Plastid Metabolic Pathways and Plastid Import Phylogenies” in the FigShare repository at https://doi.org/10.6084/m9.figshare.31541731), we frequently observe comparatively long branches for haptophyte-derived proteins in Kareniaceae, indicating accelerated evolution and, consequently, high divergence and possible loss of function. To investigate these observations further, we first generated a phylogenomic tree based on complete plastid genomes from red algae and taxa with complex red algal plastids, including the two kareniacean plastid genomes generated in this study (*K. ballantinum* and *T. helix*) and the plastid genome of *K. veneficum*. As shown by the phylogeny based on 96 ptDNA-encoded genes (Fig. 4), 2 kareniaceans carrying haptophyte plastids (*K. veneficum* and *T. helix*) form distinctly longer branches than any of the 15 haptophyte taxa with available ptDNA sequences. This suggests an accelerated mutation rate in the plastids within the dinoflagellate hosts compared to their free-living counterparts. We additionally reconstructed the phylogenies for all 96 genes separately, eliminated the trees where *K. veneficum* and/or *T. helix* sequences were missing, as well as those where the haptophyte plastid clade (haptophytes + Kareniaceae) was paraphyletic with respect to rhodophytes (to preserve the same outgroup for each tree in the analysis), and quantitatively compared the branch lengths on the remaining 21 singlegene trees. We observed a significantly increased branch length for both *K. veneficum* and *T. helix* (Mann–Whitney *U-*test; *P* < 0.00001 for *K. veneficum*; *P* = 0.00016 for *T. helix*) as compared to haptophytes and also a significant difference between the two kareniacean lineages (Mann–Whitney *U-*test; *P* = 0.00064) in favor of the former (Fig. S3). In contrast to *K. veneficum* and *T. helix*, the branch length of *K. ballantinum* does not differ from *Gephyrocapsa* spp., likely reflecting the relatively recent establishment of its plastid. Similarly, the longer branch length of *K. veneficum* relative to *T. helix* indicates a longer time span since plastid establishment in *K. veneficum* than in *T. helix*, although both plastids were taken up prior to the current *K. ballantinum* plastid.

To investigate whether the length of time since plastid establishment also relates to increased genomic structural change in these kareniacean lineages, we compared their plastid genome size and gene content. The plastid genome of *K. veneficum* is substantially inflated in size, while also being reduced in coding content (143.0 kbp, 108 genes) in comparison to those of known haptophytes (e.g., 105.3 kbp, 144 genes in *Gc. huxleyi,* and 104.5 kbp, 145 genes in *Chrysochromulina tobinii*), further supporting a potential decrease in genome stability (Gabrielsen et al. 2011; Hovde et al. 2014). For the *T. helix* plastid genome, the reduction of coding content is much less pronounced than *K. veneficum* at 130 genes, but the expansion of intergenic regions is already noticeable, as the incomplete contig we obtained is 138.8 kbp.

In contrast to circular haptophyte plastid genomes, the ancestral peridinin plastid genome is fragmented into numerous so-called minicircles, encoding for one to three genes (Nisbet et al. 2004). One can speculate that this unusual plastid genome organization is associated with functional divergence of plastid genome maintenance mechanisms (as reflected, e.g., in plastid RNA editing), in turn resulting in a decreased ability to support conventionally organized genomes, such as the one from haptophytes. Overall, the frequent plastid replacements in this group, in combination with the seemingly increased mutational rates of the plastid genomes inside these dinoflagellate hosts, indicate a potential inability of the dinoflagellates to maintain the foreign plastids long-term, therefore necessitating relatively frequent replacement. Alternatively, we consider it possible that the elevated rate of evolution of plastid-encoded genes in haptophyte-derived plastids in Kareniaceae is not deleterious, but is rather a feature of co-evolution with plastid genes transferred to and now encoded in the nucleus. One can imagine that, since these nucleus-encoded, plastid-targeted genes are interacting with genes encoded in the plastid, selection favors mutation-based adaptation of the latter to facilitate interaction. Indeed, in several phylogenetic trees we reconstructed for such nuclear-encoded, plastid-targeted genes, we observed longer branches for transferred genes present in Kareniaceae compared to the donor taxa (see “Plastid Metabolic Pathways and Plastid Import Phylogenies” in the FigShare repository at https://doi.org/10.6084/m9.figshare.31541731), indicating elevated evolutionary rates since their transfer from the plastid. However, since these long-branching sequences usually do not constitute the only copy found in the respective species (with the original dinoflagellate homolog also frequently being present), it is possible that their accelerated evolution may reflect relaxed selective pressure resulting from functional redundancy.

## Conclusions

In this study, we performed an in-depth transcriptomic analysis of two representatives of the family Kareniaceae, a lineage of dinoflagellates known for replacing its original peridinin plastids with haptophyte-derived organelles. In *Gt. stigmatica*, described as the first extant kareniacean carrying a peridinin plastid, we did not identify any clear candidate for haptophyte-derived plastid-associated gene transfer, and the overall number of gene transfers from haptophytes was distinctly lower than in haptophyte plastid-bearing Kareniaceae. We hypothesize that *Gt. stigmatica* has potentially interacted with haptophytes at some point in their evolutionary history, either as food or even as transient endosymbionts, but has probably never maintained a permanent haptophyte plastid. This provides strong support to a model in which the last common ancestor of Kareniaceae carried a peridinin plastid, which has been subsequently replaced with haptophyte-derived plastids in at least two of its descendant lineages independently.

Our analysis of the transcriptome and plastid genome of *Karlodinium ballantinum* revealed the presence of a haptophyte-derived plastid with clearly distinct origin, unusual for most kareniacean lineages. The *K. ballantinum* plastid genome is nearly identical to that of the haptophyte *Gc. huxleyi*, suggesting very recent plastid acquisition. At the same time, we found no *Gc. huxleyi*-derived genes that would represent a clear case of EGT from the current plastid within the nucleus of *K. ballantinum*. This observation challenges the general paradigm stating that the establishment of an endosymbiotic organelle is associated with large-scale EGT and also corresponds to the small numbers of EGT detected in the hosts of transient plastids (Karnkowska et al. 2023; Sørensen et al. 2023). Our findings show that the already existing plastid maintenance system can support plastids of different origins, enabling the establishment of a new permanent endosymbiotic organelle without the need for acquiring additional genes from its donor.

## Materials and methods

### Acquisition and cultivation of dinoflagellate strains

The environmental strain of *T. helix* AmTk649 was obtained from a sample of surface seawater, taken in September 2020 from Mutsu Bay, Aomori Prefecture, Japan (40°55′ N, 141°07′ E). The environmental strain *K. ballantinum* HrKl655 was obtained from a sample of surface seawater, taken in September 2020 from the coast of Etajima Island, Hiroshima Prefecture, Japan (34°14′ N, 132°29′ E). The strain *Gt. stigmatica* mdd472-kt, established in previous work (Takahashi et al. 2019) and deposited at the NIES culture collection (National Institute for Environmental Studies, Japan), has been maintained at the University of Tokyo and used in this study. Clonal monoeukaryotic cultures of all strains were obtained from manually picked single cells and maintained on IMK/2 medium (variant of Daigo IMK medium; Nihon Pharmaceutical Co., Ltd., Japan), in 20 °C, under 14 h/10 h light–dark cycle for *Gt. stigmatica* and in 23 °C, under 12 h/12 h light-dark cycle for *K. ballantinum* and *T. helix*.

### Plastid genome sequencing, assembly, and annotation

Genomic DNA was extracted from the clonal cultures of *K. ballantinum* and *T. helix* using the DNeasy Plant Mini Kit (QIAGEN), followed by whole genome amplification using the REPLI-g Single Cell Kit (QIAGEN) according to the manufacturer's instructions. Sequencing libraries were prepared using the VAHTS Universal Pro DNA Library Prep Kit for Illumina (Vazyme). Genome sequencing was performed at the University of Tsukuba, Japan, using the Illumina HiSeq X machine (Illumina).

Quality control of the obtained reads was carried out using FastQC v0.11.9 (Andrews 2010). Removal of Illumina adapter sequences and reads of low quality was performed using Trimmomatic v0.39 (Bolger et al. 2014). Preliminary genome assembly was performed with SPAdes v3.15.2 (Prjibelski et al. 2020). Contigs derived from the plastid genomes were identified with BLASTN (Altschul et al. 1990) using queries from published plastid genome sequences of *K. veneficum* (NCBI accession no. JN039300), *C. tobinii* (NCBI accession no. KJ201907), and *Gc. huxleyi* (formerly *Emiliania huxleyi*; NCBI accession no. AY741371). The longest identified contig in each dataset was extracted and used as a seed sequence for NOVOPlasty v4.3.3 assembly (Dierckxsens et al. 2017), which yielded a successfully assembled circular ptDNA contig of 105,613 bp in the case of *K. ballantinum*, and a linear (incomplete) ptDNA contig of 138,828 bp in the case of *T. helix*. Annotation of the plastid genome sequences was performed using Live Annotate & Predict: Annotate from Source function in Geneious Prime v2023.0.4 (https://www.geneious.com), using the three aforementioned published plastid genomes as reference, and corrected manually. Annotated plastid genome sequences have been deposited in the NCBI GenBank database under accession numbers PZ117046 (*K. ballantinum*) and PZ117047 (*T. helix*). Mapping of transcriptomic reads onto the plastid genome sequence of *T. helix* was performed with bowtie v2.4.4 (Langmead and Salzberg 2012) using default endtoend alignment settings, and the obtained SAM file was processed for viewing and coverage analysis using samtools v1.20 (Danecek et al. 2021). Plastid genome maps were generated using OGDRAW v1.3.1 (Greiner et al. 2019).

### Transcriptome sequencing, assembly, and protein prediction

Total RNA was extracted from the culture material of *Gt. stigmatica* using TRIzol protocol (Invitrogen) and from *K. ballantinum* using NucleoSpin RNA Clean-up kit (Macherey-Nagel GmbH & Co) with an additional DNA removal step using TURBO DNA-free Kit (ThermoFisher Scientific). The cDNA library from *Gt. stigmatica* was prepared using KAPA Stranded mRNASeq Kit (Roche) and from *K. ballantinum* using Illumina TruSeq Stranded mRNA kit (Illumina). All procedures were done following the manufacturers′ instructions.

Transcriptome sequencing was performed by external companies, using a NextSeq 500 machine (Illumina), yielding 151 bp long paired-end reads in the case of *Gt. stigmatica*, and using a HiSeq machine (Illumina), yielding 101 bp long paired-end reads in the case of *K. ballantinum*. Quality control of the obtained reads was carried out using FastQC v0.11.9 (Andrews 2010). Removal of Illumina adapter sequences and reads of low quality was performed using Trimmomatic v0.39 (Bolger et al. 2014). Transcriptome assembly was carried out using Trinity v2.15.1 (Haas et al. 2013), yielding 103,859 contigs for *Gt. stigmatica* and 155,264 contigs for *K. ballantinum.* The completeness of the assemblies was assessed with BUSCO v5.5.0 (Manni et al. 2021). Coding DNA sequence prediction was performed with TransDecoder v5.5.0 (Haas, BJ; https://github.com/TransDecoder/TransDecoder). The assembled transcripts and predicted peptides for *K. ballantinum* and *Gt. stigmatica* are available at https://doi.org/10.6084/m9.figshare.31529722.

### Ortholog group reconstruction, single-gene phylogeny, and tree sorting

Orthogroups for predicted proteins from the transcriptomes of *Gt. stigmatica* and *K. ballantinum* were reconstructed using OrthoFinder v2.5.4 (Emms and Kelly 2019) with the input of 103 proteomes of dinoflagellates and haptophytes, reconstructed from the published transcriptomic datasets from 11 strains of Kareniaceae, 61 strains of other dinoflagellates, and 31 strains of haptophytes. Phylogenies of all 254,267 obtained orthogroups were reconstructed by FastTree (Price et al. 2010), embedded within the OrthoFinder package, and are further referred to as local trees.

Single-gene local trees containing *Gt. stigmatica* (27,839 trees) and/or *K. ballantinum* sequences (42,728 trees) were filtered using an ETE3-based (Huerta-Cepas et al. 2016) in-house Python script (https://github.com/ProtistomicsLab/scripts-public/blob/main/kocur.py) to identify trees containing a monophyletic clade of *Gt. stigmatica* and haptophyte sequences, or a monophyletic clade of *K. ballantinum* and Noelaerhabdaceae sequences. The results were assessed manually, and for each positive hit, protein sequences from *Gt. stigmatica* or *K. ballantinum* were extracted and used as queries for BLASTP against a custom database of published transcriptomic data, and the results were parsed into fasta sequences at various *e-*value cutoffs, depending on their size (from e-5 to e-40), to reconstruct global ortholog groups. For each recovered group, sequences were aligned via mafft v7.525 (Katoh and Standley 2013), alignments were trimmed using trimAl v1.4 (Capella-Gutiérrez et al. 2009), and the global phylogenetic trees were calculated with RAxML v8.2.12 under the LG + G4 substitution model (PROTGAMMALG4X) with 100 rapid bootstrap replicates (Stamatakis 2014). Functions of the proteins of interest were identified using BLASTP against NCBI-nr database, and using the online InterProScan service (Blum et al. 2025).

### Plastid-associated single-protein phylogenies

Orthologs of proteins of interest (see Fig. 2 for investigated pathways and systems) were selected from previously generated phylogenies, selecting at least one candidate for each eukaryotic supergroup present in the tree. These orthologs were used as BLASTP queries against a comprehensive custom protein database containing representatives from most major eukaryotic groups, with a focus on plastid-containing lineages plus selected taxa from non-plastidial lineages and RefSeq data from all bacterial phyla at NCBI (last accessed December 2017). The database was subjected to CD-HIT (Fu et al. 2012) with a similarity threshold of 85% to reduce redundant sequences and paralogs, except for the two datasets of *K. ballantinum* and *Gt. stigmatica* and the two RSD datasets created in Hehenberger et al. (2019), which were clustered at 98%. Search results of the BLASTP step were parsed for hits with an *e-*value threshold ≤1e-25 and a query coverage ≥50% to reduce the possibility of inclusion of paralogs and short sequences. For short proteins (e.g., photosystem subunits), the parsing parameters were adjusted in individual cases. The number of bacterial hits was restrained to 20 hits per phylum (for Fibrobacterota-Chlorobiota-Bacteroidota group, most classes of Proteobacteria, Planctomycetota-Verrucomicrobiota-Chlamydiota group, Spirochaetes, Actinobacteria, Cyanobacteria [unranked], and Firmicutes) or 10 per phylum (remaining bacterial phyla) as defined by NCBI taxonomy. Parsed hits of queries corresponding to the same protein were combined, deduplicated, and initially aligned with MAFFT v7.525 (Katoh and Standley 2013) using the --auto and the --reorder options; phylogenetically informative sites were identified with ClipKIT (Steenwyk et al. 2020) using default options, and initial maximum likelihood (ML) tree reconstructions were performed with FastTree v2.1.11 (Price et al. 2010) using default options. Resulting phylogenies and underlying alignments were inspected manually to remove contaminations and poor-quality sequences in several iterations, repeating the tree reconstruction as described above and continuously realigning in the AliView (Larsson 2014) alignment viewer, using the MAFFT L-INS-i algorithm. For some of the SELMA alignments (Cdc48, Hsp70, Ubi), fully supported clades containing published representatives of Kareniaceae (Lewis et al. 2024) have been extracted to reduce the size of the original alignment which was computationally prohibitive. Due to very long and variable N-terminal extensions in dinoflagellates (sometimes longer than the actual protein), we removed the N-termini of alignments with N-terminal targeting information and retained only the conserved proteins. Final, cleaned alignments were then aligned with MAFFT G-INS-i using the VSM option (--unalignlevel 0.6) (Katoh and Standley 2013) to control over-alignment. The alignments were subjected to Divvier (Ali et al. 2019) using the -divvygap option to improve homology inference before removing ambiguously aligned sites with trimAl v1.2 (-gt 0.01) (Capella-Gutiérrez et al. 2009). Finally, ML trees were calculated with IQ-TREE v3.0.1 (Wong et al. 2026), using the LG + C60 + G model and the -mwopt option (Baños et al. 2024). 100 RAxML rapid bootstraps (Stamatakis et al. 2008) were calculated and mapped to the generated trees. All phylogenies have been deposited to a FigShare repository as PDF figures and in Nexus format (see https://doi.org/10.6084/m9.figshare.31541731).

### Plastid-based phylogenomic analysis

Totally, 96 protein-coding plastid genes were selected as the input dataset for a phylogenetic analysis. Sequences for 26 taxa carrying plastids from the red algal lineage, including 5 representatives of Rhodophyta, 3 representatives of Ochrophyta, 2 representatives of Cryptophyta, 15 representatives of Haptophyta, and 1 representative of Kareniaceae (*K. veneficum*), were extracted from the publicly available records in the NCBI GenBank database. In addition, sequences for *K. ballantinum* and *T. helix* were extracted from the plastid genome assemblies obtained in this study. Unaligned sequences were filtered using PREQUAL on default settings (Whelan et al. 2018). Gene alignments were performed using MAFFT v7.505 (Katoh and Standley 2013) in global pairing (G-INS-i) mode (--*globalpair*), followed by an additional homology inference improvement step performed with Divvier (Ali et al. 2019) and trimming using trimAl v1.4 (Capella-Gutiérrez et al. 2009) using default settings with gap threshold 0.01. Alignments were concatenated using the catsequences script (https://github.com/ChrisCreevey/catsequences/) to produce a sequence matrix of 20,698 positions. The multigene phylogeny was calculated using IQTREE v2.1.0 (Minh et al. 2020) with the two-step posterior mean site frequency (PMSF) approach, which included the reconstruction of a guide tree in the first step and the final tree with the LG + C60 + G substitution model plus the -mwopt option (Baños et al. 2024) and 500 non-parametric bootstrap replicates in the second step (Wang et al. 2018).

### Plastid-based single-gene phylogeny and branch length analysis

Trimmed alignments of 21 out of 96 protein-coding plastid genes, obtained as outlined above (see the Plastid-based phylogenomic analysis subsection), were used for reconstruction of single-gene phylogenetic trees using IQ-TREE v2.1.0 (Minh et al. 2020) with the LG + C60 + G substitution model plus the -mwopt option (Baños et al. 2024), and branch support was inferred with 100 non-parametric bootstrap replicates. Branch length values for haptophyte and dinoflagellate-derived sequences were extracted using an original in-house Python script (https://github.com/ProtistomicsLab/scripts-public/blob/main/branchlen.py). Statistical analysis of the obtained data via Mann–Whitney *U-*test was performed using rstatix package v0.7.2 (https://cran.r-project.org/web/packages/rstatix/) in R v4.2.2. The data were visualized as a boxplot using packages tidyverse v2.0.0 (https://cran.r-project.org/web/packages/tidyverse/) and ggpubr v0.6.0 (https://cran.r-project.org/web/packages/ggpubr/), implemented in R v4.2.2. Phylogenies and underlying alignments have been deposited to a FigShare repository and are available at https://doi.org/10.6084/m9.figshare.31422221.

### Nuclear phylogenomic analysis

Ortholog groups for phylogenomic analysis were obtained using PhyloFisher (Tice et al. 2021). Eighty-one new dinoflagellate taxa (including two datasets for *Oxyrrhis marina*) were added to the PhyloFisher database, 7 of which replaced datasets of the same species or a congener, increasing taxon coverage from 8 (including *P. marinus*) to 82 taxa. Datasets were filtered using PREQUAL (Whelan et al. 2018), aligned using MAFFT v7.505 (G-INS-i) (Katoh and Standley 2013), followed by an additional improvement step performed with Divvier (Ali et al. 2019). Trimming was performed using trimAl v1.4 (Capella-Gutiérrez et al. 2009). These four steps were performed manually with parameter settings as described in Tice et al. (2021). Single-gene trees were inferred using the “rapid bootstrap analysis and search for the best scoring ML tree in one” option (-f a) in RAxML v8.12.12 (Stamatakis 2014) under the PROTGAMMALG4X model with 100 distinct starting trees (-N 100). Resulting trees were rendered using forest.py (Tice et al. 2021) and manually curated using parasorter.py (Tice et al. 2021) for determining best orthologs and excluding contamination. Finally, using PhyloFisher (Tice et al. 2021), a matrix of 73,923 amino acids in length was concatenated from 228 proteins, which were covered by at least 10% of the 85 datasets (including two chromerid outgroup taxa). The multigene phylogeny was calculated from this matrix using IQ-TREE v2.1.0 (Wang et al. 2018; Minh et al. 2020) in a twostep PMSF approach, inferring a guide tree in the first step and the final tree in the second step using the LG + C60 + G substitution model and the -mwopt option (Baños et al. 2024), with 500 non-parametric bootstrap replicates. The untrimmed and trimmed alignments underlying the single-gene trees and the final tree, and the corresponding alignment are available at https://doi.org/10.6084/m9.figshare.31529530 and https://doi.org/10.6084/m9.figshare.31528447.

## Supplementary Material

msag166_Supplementary_Data

## Data Availability

Plastid genome sequences obtained in this study are available in the NCBI GenBank repository under accession numbers PZ117046 (*K. ballantinum*) and PZ117047 (*T. helix*). All novel transcriptomic data and predicted peptide sequences from *K. ballantinum* and *Gt. stigmatica* are available in the FigShare repository at https://doi.org/10.6084/m9.figshare.31529722. Sequence alignments and phylogenetic trees obtained in this study are available in the FigShare repository at https://doi.org/10.6084/m9.figshare.31541731, https://doi.org/10.6084/m9.figshare.31421609, https://doi.org/10.6084/m9.figshare.31410999, https://doi.org/10.6084/m9.figshare.31528447, https://doi.org/10.6084/m9.figshare.31529530, and https://doi.org/10.6084/m9.figshare.31422221. All raw sequencing data underlying this study will be shared upon request to the corresponding author.
