# Supplementary material for: Recent plastid replacement in Karlodinium ballantinum (Kareniaceae, Dinoflagellata) challenges the paradigms of endosymbiotic gene transfer
Source: Mol Biol Evol. 2026 Jul 7;43(7):msag166. doi: 10.1093/molbev/msag166 (PMC13394690; doi:10.1093/molbev/msag166)
Supplement: msag166_Supplementary_Data [file msag166_supplementary_data.zip › Maciszewski_revised_Table_S2.pdf]

Table S2. BUSCO v5.4.3 completeness scores for the available transcriptomic datasets from Kareniaceae (obtained using eukaryota_odb10 as reference BUSCO database).

**Taxon**	**Complete (single)**	**Complete (duplicated)**	**Fragmented**	Missing	**CS+CD+F**
Gertia stigmatica	58.4	7.8	9.0	24.8	**75.2**
Karlodinium ballantinum	56.5	23.9	5.1	14.5	**85.5**
Karenia brevis CCMP2229	61.2	3.5	14.5	20.8	**79.2**
Karenia brevis SP1	63.5	3.1	11.4	22.0	**78.0**
Karenia brevis Wilson	63.5	3.5	12,.2	20.8	**79.2**
Karenia mikimotoi	65.9	6.7	9.0	18.4	**81.6**
Karenia papilionacea	59.2	10.2	12,5	18.1	**81.9**
Ross Sea Dinoflagellate	31.0	46.3	9.0	13.7	**86.3**
Karlodinium armiger	62.7	7.8	11.0	18.5	**81.5**
Karlodinium micrum	63.9	6.3	11.0	18.8	**81.2**
Karlodinium veneficum	56.9	3.9	12.5	26.7	**73.3**
Takayama helix	71.0	6.3	6.3	16.4	**83.6**
