# Supplementary material for: Recent plastid replacement in Karlodinium ballantinum (Kareniaceae, Dinoflagellata) challenges the paradigms of endosymbiotic gene transfer
Source: Mol Biol Evol. 2026 Jul 7;43(7):msag166. doi: 10.1093/molbev/msag166 (PMC13394690; doi:10.1093/molbev/msag166)
Supplement: msag166_Supplementary_Data [file msag166_supplementary_data.zip › Maciszewski_revised_Table_S4.pdf]

Table S4. Prevalence of *thiG* gene among extant haptophytes.

Species/strain	***thiG* present?**
Chrysochromulina parva	+
Chrysochromulina tobinii	+
Diacronema lutheri	-
Gephyrocapsa ericsonii	+
Gephyrocapsa huxleyi	+
Gephyrocapsa muellerae	+
Gephyrocapsa oceanica	+
Gephyrocapsa parvula	+
Isochrysis galbana	+
Pavlomulina ranunculiformis	-
Pavlova sp. NIVA-4/92	-
Phaeocystis antarctica	-
Phaeocystis globosa	-
Phaeocystis rex	-
Prymnesium parvum	+
Prymnesium polylepis	+
Tisochrysis lutea	+
