# Supplementary figures and images for: Recent plastid replacement in Karlodinium ballantinum (Kareniaceae, Dinoflagellata) challenges the paradigms of endosymbiotic gene transfer
Source: Mol Biol Evol. 2026 Jul 7;43(7):msag166. doi: 10.1093/molbev/msag166 (PMC13394690; doi:10.1093/molbev/msag166)

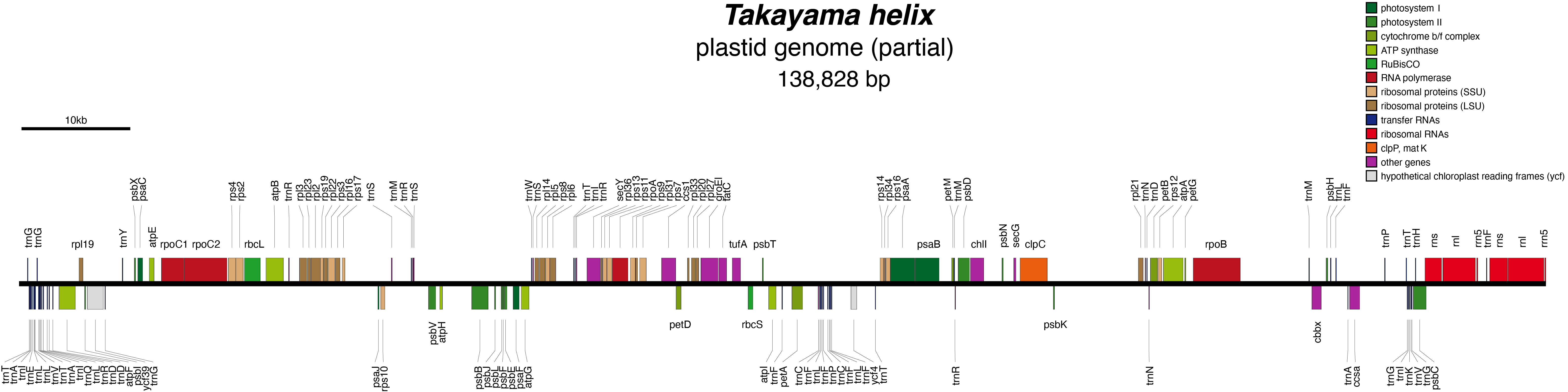

Supplement: msag166_Supplementary_Data [file msag166_supplementary_data.zip › Maciszewski_final_Figure_S2.tif]

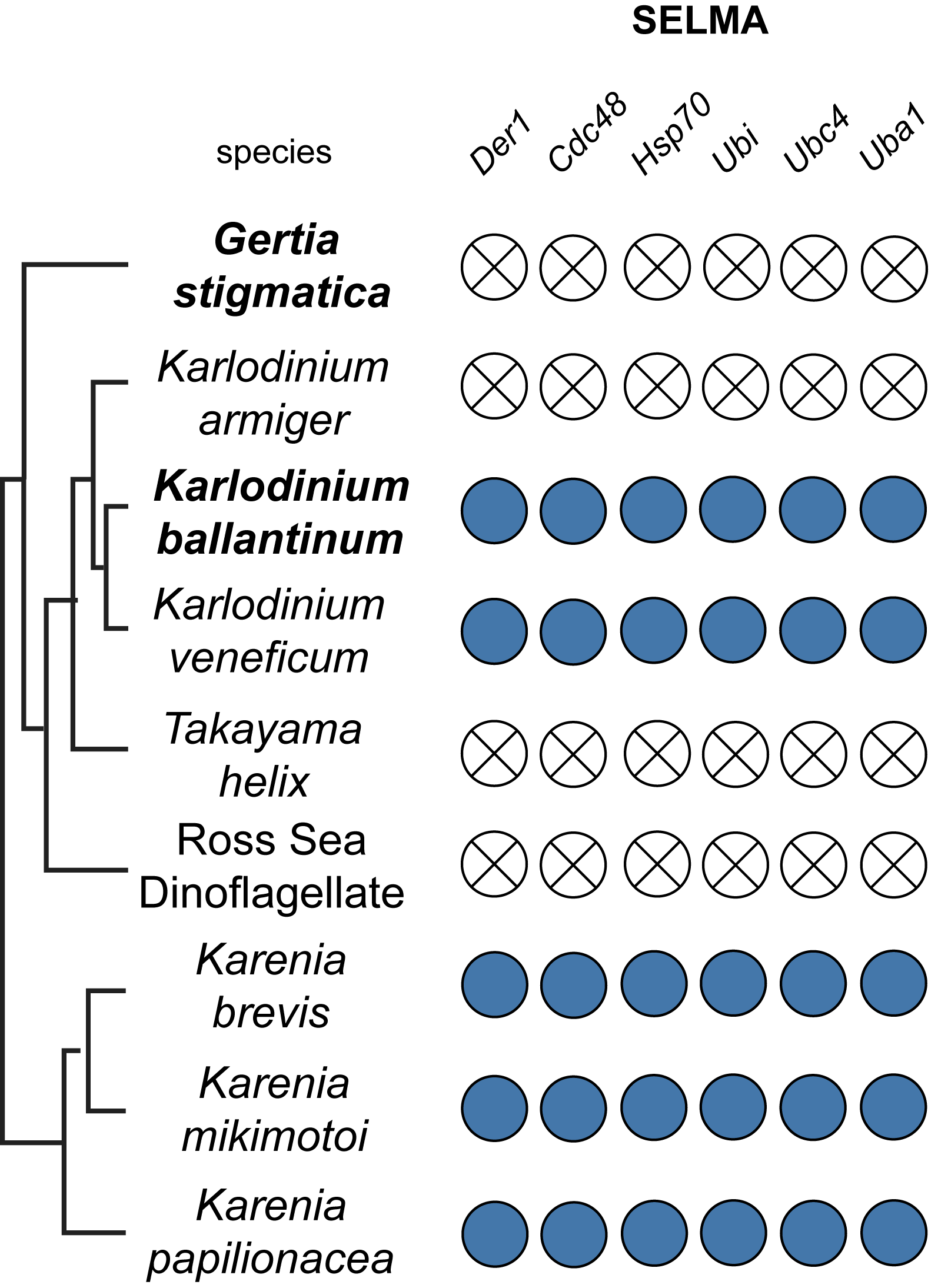

Supplement: msag166_Supplementary_Data [file msag166_supplementary_data.zip › Maciszewski_revised_Figure_S1.tif]

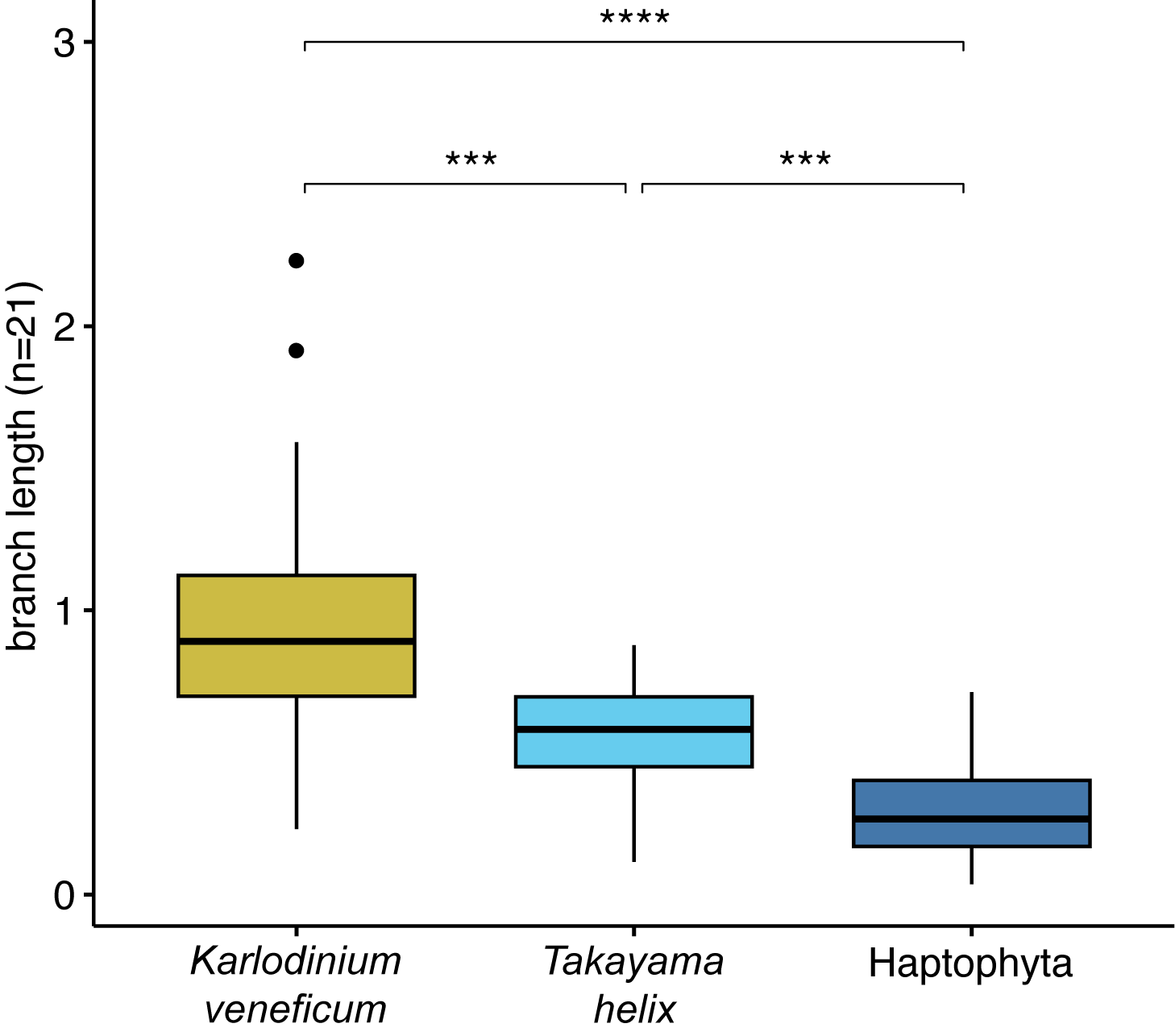

Supplement: msag166_Supplementary_Data [file msag166_supplementary_data.zip › Maciszewski_revised_Figure_S3.tif]
